# The Development of Sustainable Engineering with PjBL during the COVID-19 Pandemic †

**DOI:** 10.3390/ijerph20054400

**Published:** 2023-03-01

**Authors:** Victor Takashi Hayashi, Reginaldo Arakaki, Felipe Valencia de Almeida, Wilson Vicente Ruggiero

**Affiliations:** Polytechnic School, University of São Paulo, Av. Prof. Luciano Gualberto, 908, Butantã 05508-010, SP, Brazil

**Keywords:** engineering education, sustainable development goals, remote lab, project based learning

## Abstract

Sustainable Engineering education must provide cyber-physical and distributed systems competencies, such as the Internet of Things (IoT), to contribute to the Sustainable Development Goals (SDG). The COVID-19 pandemic caused profound impacts arising from a traditional on-site teaching model rupture and demanded distance learning for engineering students. In this context, we considered the following Research Questions (RQ): How can Project Based Learning (PjBL) be applied in hardware and software courses from the Engineering curriculum to foster practical activities during the COVID-19 pandemic? Is the student performance in the fully remote offering comparable to the face-to-face offering? (RQ1); Which Sustainable Development Goals are related to the Engineering students’ project themes? (RQ2). Regarding RQ1, we present how PjBL was applied in first-, third- and fifth-year Computer Engineering Courses to support 31 projects of 81 future engineers during the COVID-19 pandemic. Student grades in a Software Engineering course indicate no relevant differences between student performance in remote and face-to-face offerings. Regarding RQ2, most Computer Engineering students from the Polytechnic School of the University of São Paulo in 2020 and 2021 decided to create projects related to SDG 3—Good Health and Well-being, SDG 8—Decent Work and Economic Growth and SDG 11—Sustainable Cities and Communities. Most projects were related to health and well-being, which was an expected behavior according to how health issues were brought into highlight during the pandemic.

## 1. Introduction

Social distancing and the consequences for education are subjects for continuous reflections on how we evolve teaching techniques and methodologies. This holds for Engineering students: they must learn concepts, exercise them, and discuss their applications in a real-life scenario. The situation caused by the COVID-19 pandemic created many touching points in education themes, especially regarding virtual interactions.

In this paper, we describe some study cases: the first is from an introductory course for first-year Engineering students. The second one regards the Digital Electronics Lab II applied for third-year students; the third one describes a scientific initiation, a short-term research project for a beginner Engineering student; the fourth report describes a Software Engineering Lab for fifth-year students. The last study case involved an Engineering Capstone Project where technologies and methods were presented by advisors and co-advisors to allow student teams to obtain consistent technical and business orientations.

Some principles were fundamental to developing these education study cases:

- Principle 1: Collaboration teams with professors, students, technicians, and staff teams discussing the importance of the traditional way to teach [1];
- Principle 2: Real-time data and actions using electronic sensors, actuators and other digital tools to improve the virtual platform to immerse students in their activities inside the laboratory instruments. In this case, considering four roles: students in pairs, each in their home; professor to assist, help with and monitor learning results; the laboratory pieces of equipment and components accessed remotely; and technicians for supporting all activities [2,3];
- Principle 3: Similar to the previous item, the Internet of Things was fundamental here. Students created innovative projects using various types of sensors and actuators, Internet connections, cloud processing resources to support smartphone applications as evidence of digital transformation results, and providing an experience resembling an augmented reality [4];
- Principle 4: Application of remote laboratory for simple experiments would be helpful, but solving practical problems was the mainstream directive to engage teams. They were strongly oriented to search for and obtain real situations of people in their day-by-day routines. This line of thought is aligned to the Problem Based Learning method [5];
- Principle 5: Value proposition was one of the main aspects of inspiring, creating, and executing learning activities. As a result, all projects had to show what and how to add value for people [2].

**Figure 1:**
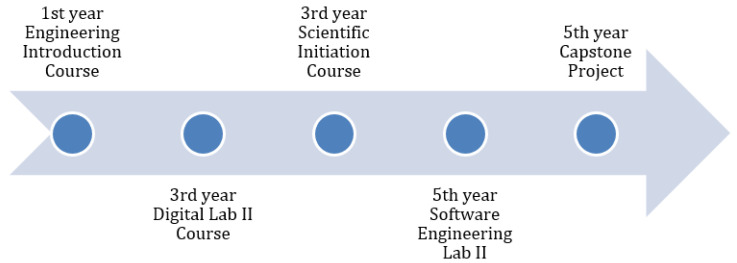
PjBL initiatives during social distancing for Computer Engineering in 2020.

Figure 1 presents a timeline of the learning initiatives presented. All Lines of Thought cited here created engagement from students. In the laboratory activities, they were motivated by using canvas to improve the value proposition of their projects [6]. Significant results in terms of project quality by applying immersive activities using IoT in a remote laboratory environment. In their final course work, innovative teams’ awards are evidence of marks obtained by combining these principles. Additionally, we supported the Engineering Introduction Course for first-year students, Digital Lab for third-year students, and Software Engineering Lab for fifth-year students in 2021.

A total of 81 students from the first (52), third (14 + 1) and fifth-years (13 + 1) could develop projects related to the United Nations Sustainable Development Goals. It is interesting that the students chose these themes independently (i.e., the teachers did not suggest the project themes).

This article is an extended version of our paper published at the International Conference on Active Learning in Engineering Education (PAEE/ALE 2021) [7]. Compared to the original paper with reports of disciplines from 2020, we added the results of 2021, the analysis of project themes related to the Sustainable Development Goals, and the student performance comparison of face-to-face and fully remote Software Lab course offerings.

The text is organized as follows: Section 2 describes the research contribution considering the literature, the methodology is presented in Section 3, and Section 4 contains the results regarding project themes. A comparison to related work and the responses to research questions are presented in Section 5. We conclude with final comments in Section 6.

In this work, we considered two research questions. These Research Questions are supported by the existing literature. A research gap presented in the literature is to incorporate practical exercises (sensor and actuator-based activities) in Project Based Learning (PjBL) courses [8]. Considering the limitations imposed by the COVID-19 pandemic, continuous assessment of hybrid and remote classes is a must to investigate different approaches [9]. Embedding sustainability in Final Degree projects and identifying the relationships between Sustainable Development Goals (SDG) and Engineering Education worldwide are also some research gaps [10]. Another research gap is related to the investigation of educational units’ impacts on SDGs [11].

**Research Question 1.** *How can Project Based Learning (PjBL) be applied in hardware and software courses from the Engineering curriculum to foster practical activities during the COVID-19 pandemic? Is the student performance in the fully remote offering comparable to the face-to-face offering?*

**Research Question 2.** *Which Sustainable Development Goals are related to the Engineering student’s project themes?*

## 2. Research Contribution

Project-Based Learning (PjBL) is a pedagogy that believes that the students can learn better by interacting with real-world challenges (learning by doing), resulting in an active knowledge construction [12]. The PjBL approach contrasts with the traditional classroom approach, where the students spend most of the time hearing the teacher and taking class notes more passively. Learning starts with a driving question, which students need to solve. Students and teachers work together to find the solution in the solving process. Students have greater autonomy in managing their time and activities, while the teachers work as learning facilitators.

The PjBL approach is not new in the literature, but its use was intensified during the pandemic as a way to mitigate the problems of emergency remote teaching [13]. Edy et al. [14] carries out an impact analysis of PjBL on online learning. The authors recognize the advantages of this approach and point to the need better to understand students’ motivation during the online learning process. Pérez-Sánchez et al. [15] presents a case study of a PjBL application in Fluid-Mechanical Engineering. The authors had to adapt their classes for emergency remote teaching using different tools. Classes were offered to 136 students, of which 76.6% who answered the survey preferred the PjBL classes. Parhusip et al. [16] experimented using Solidworks software with PjBL online learning classes. The results show that the PjBL application increased student activity during classes and improved learning. Halaweh [17] debates the use of traditional tools and approaches during the pandemic. The PjBL assessment is presented as a better way to evaluate students learning without concern about detecting cheating cases, which most classes cannot fully eliminate.

We can also find many works of literature describing the relationship between education and sustainable development. Maybe, the biggest of them is the United Nations Educational, Scientific and Cultural Organization (UNESCO) Education for sustainable development (ESD), which aims to give learners of all ages the knowledge, skills, values and agency to address interconnected global challenges [18].

It was also possible to find works that apply the PjBL in the sustainable development context. Corvers et al. [19] show the advantages of adopting the PjBL for sustainable development but also point to the challenges of this approach. Nation [20] presents a similar discussion and shows a significant challenge to actively involve students in research with communities. There are also case studies that show how applying the PjBL helped the students better understand sustainability and the global challenges [21,22,23].

The present study contributes to the body of knowledge by describing how the PjBL was applied in the Computing Engineering remote courses during the COVID-19 pandemic at a Brazilian University. To the best of authors’ knowledge, it is one of the first works that show the relationship between projects’ themes chosen by the students and the United Nations Sustainable Development Goals (SDG).

Another difference between our work and other works found in the literature is in scope. While most works realize a high-level context analysis about applying PjBL or present a single case study, this article has a multiple case study. Five different case studies are presented where we explored the use of PjBL in different classes for different students and even out-of-class contexts.

## 3. Methodology

This section presents the remote laboratory and online whiteboard tools, the data collection method and the methodology applied in the Computer Engineering courses.

### 3.1. Tools

This subsection describes the remote laboratory and online whiteboard tools used in this work.

#### 3.1.1. Remote Lab

**Figure 2:**
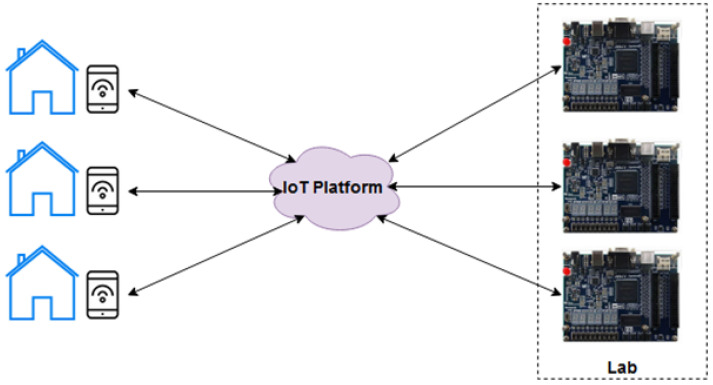
LabEAD high-level concept. Students perform activities from home by interacting with mobile devices and the FPGA boards are accessed using an IoT platform (as illustrated by the communication arrows).

LabEAD (Remote Lab for Engineering) [1,5] is a series of initiatives to make remote teaching of laboratory subjects feasible for Engineering courses. In this paper, we present the LabEAD in a Digital Laboratory course context. Figure 2 presents a high-level concept of the LabEAD concept. Each student in their residence can access the laboratory benches by connecting to an IoT platform using their smartphones and computers.

**Figure 3:**
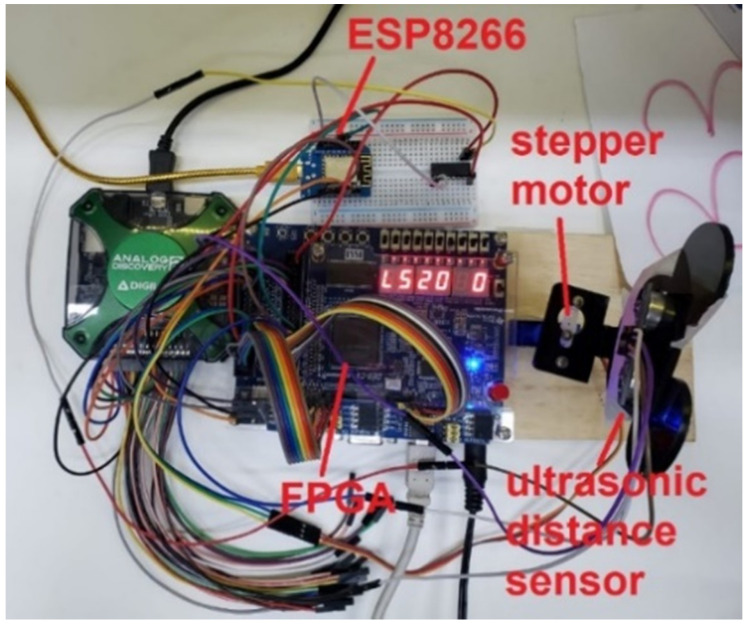
LabEAD physical assembly.

We decided to use Blynk IoT Platform (product from Blynk company, New York, NY, USA) as the IoT cloud platform, but any other platform could be used if the required changes are made in the source code. Each student needed to download the Blynk app from the Google Play/App Store and then use it to send commands to the laboratory bench. An IoT architecture was built in the laboratory using the FPGA board, which is the core of the Digital Laboratory course, an ESP8266 component, which has a Wi-Fi module included and some input/output expansion devices. The physical assembly is presented in Figure 3.

The project source code is available at: https://github.com/vthayashi/labead-labdig (accessed on 1 February 2023).

#### 3.1.2. Online Whiteboard

**Figure 4:**
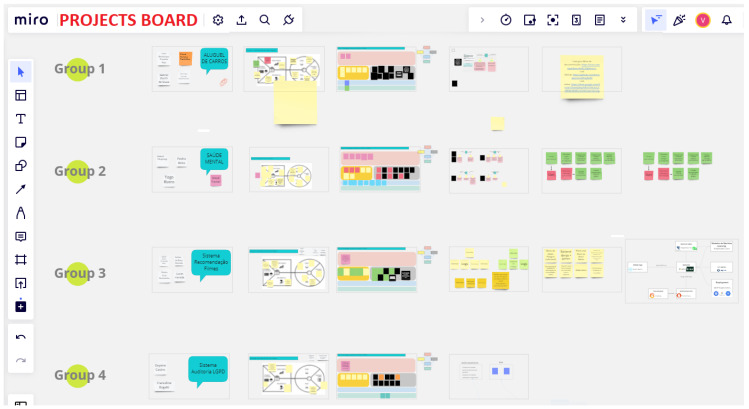
Example of using Miro in class for fifth-year Computer Engineering students.

Miro is an online whiteboard tool that is used for collaborative projects. In our latest works [24], we explored the use of Miro in a remote educational context, in which students could actively interact with the teacher without the need to turn on their webcams or microphones, respecting their privacy. Figure 4 shows an example of a Miro board, which allows students to interact in the class using their mouse cursors.

### 3.2. Data Collection

This subsection describes the rationale of the data collection method used to gather data to support the answers to the proposed research questions.

#### 3.2.1. Participants

Students who participated in the PjBL courses during the COVID-19 pandemic were from the first-, third- and fifth-years. They were students from the Computer Engineering course at the Polytechnic School of the University of São Paulo, Brazil.

#### 3.2.2. Student Performance

The student performance was compared based on the student final grades in the Software Engineering Laboratory course offerings of 2019 (face-to-face, 10 students), 2020 (hybrid, 13 students) and 2021 (fully remote, 11 students) from a total of 34 students.

#### 3.2.3. Student Projects

Regarding the Digital Electronics Lab offered in 2020, the students’ project descriptions were collected with one questionnaire conducted in the final class, with 11 out of the 25 students. The questions are listed below.

- Provide the name of your project;
- Provide the summary of your project.

The project descriptions from the other courses were obtained from the specification and results included in the Miro tool.

### 3.3. Study Cases

The study cases of first, third and fifth-year Computer Engineering courses are presented in this subsection.

#### 3.3.1. First Year: Engineering Introduction Course

The Engineering Introduction Course is an introductory course for first-year Computer Engineering students. The objective is to provide the students with Engineering project management methods such as agile development methods. The main deliverable is a project performed in groups of four students with themes of free choice. In 2021, the students used the Miro tool during classes to collaborate in the specification, development and presentation of their projects. The professors assumed the role of counselors, and some Graduate students and Industry Engineers provided technical workshops to support the projects.

Some interesting project themes are listed below:

- Academic activities scheduler;
- Sleep health assistant;
- Shopping list app;
- Academic Tests app;
- Smart Medicine box;
- News Listener app;
- Smart Agro platform;
- Event Promoter app;
- Educational Game for kids;
- Restaurant Scheduler;
- Beekeeping assistant;
- Water management app;
- Smart Home Locker.

#### 3.3.2. Third Year: Digital Electronics Lab

Digital Laboratory II is a discipline offered to third-year students. Students work in groups with a complete hardware development cycle using a hardware description language (HDL) and an FPGA board.

The cycle starts with the project specification, where they need to identify which hardware components are required to implement the requested circuit, such as logic gates, registers, multiplexers, and other components. After the specification, they need to write the project source code with the HDL and then test it using simulation tools like ModelSim. For the first half of the cycle, students must make a report before the class, submitting the report to the professor with the results. In the class, they continue the cycle, compiling the code using the FPGA board development proprietary software, generating an output file called bitstream, and then the bitstream is loaded into the FPGA board, making the FPGA behave as the circuit described. For the last part, the students must test the circuit, sending different input signals to the FPGA and checking if the output signals are correct. If any inconsistency is verified, they need to debug the project, which can take much of the class time.

In the 2020 offering, we had to adapt the course to use the LabEAD, allowing students to interact with the FPGA board and other components, such as sensors and actuators, while respecting social distancing protocols (due to the COVID-19 pandemic). That was possible with teachers, MSc students as monitors, course students, whom acted as stakeholders and the laboratory technical personnel. In addition, we used Google Meet as the videoconference tool and AnyDesk as the remote access tool to permit the students remote access to the laboratory computer machines.

The last half of the course classes are always dedicated to a project. The groups freely choose ideas related to the course concepts. The LabEAD allowed the students to integrate components in their residences with the FPGA board in the lab, giving more variability and possibilities to the projects.

The following subsections present the project descriptions provided by the students.

**Figure 5:**
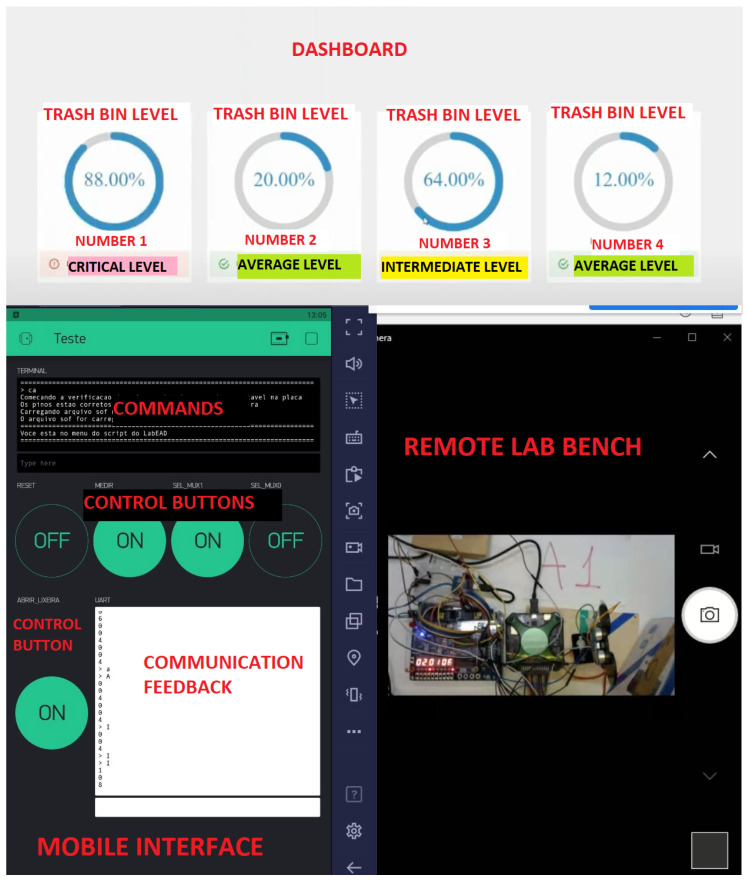
Smart Trash Bin project.

Smart Trash Bin: We worked on constructing an intelligent trash can, which informed a Control Center of its capacity. The project aims to control the filling of different dumps and facilitate truck collection. Projects of a similar scope were implemented in different locations giving the local city hall a 50% cost reduction. Figure 5 depicts the dashboard (top) and the mobile interface (bottom left), and the remote laboratory bench (bottom right).

**Figure 6:**
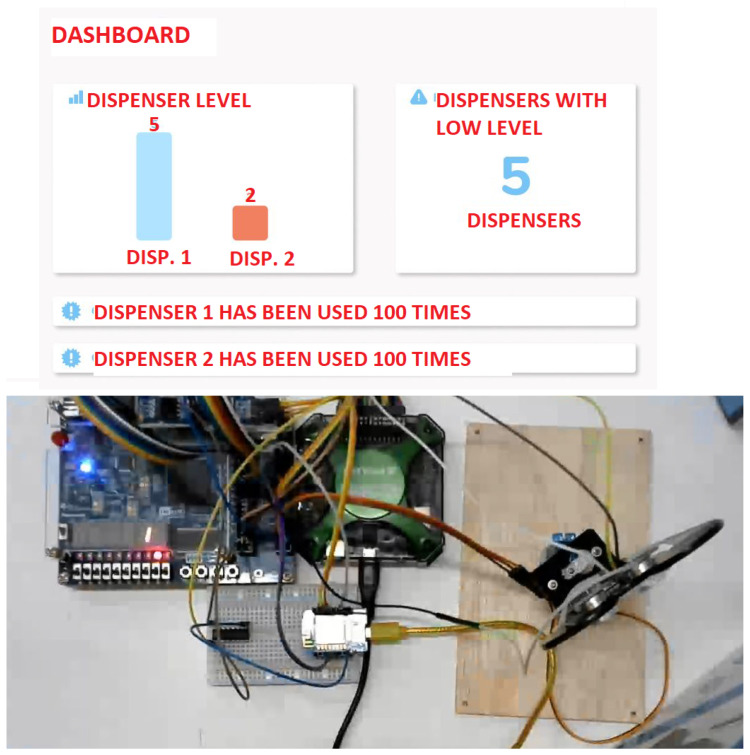
Automatic Gel Dispenser project.

Automatic Gel Alcohol Dispenser: The theme chosen by the group considers the pandemic situation in which the world currently finds itself. In this situation, two attitudes can reduce the transmission of the virus: the constant use of alcohol gel to sanitize hands and the reduction of contact with objects that can come into contact with other people. A conflict between the two attitudes can be noted, since the use of alcohol in gel implies contact with the bottles containing the liquid, which can reduce the effect of these attitudes. With this problem in mind, the group proposed an automatic gel alcohol dispenser that eliminates physical contact with the equipment. This apparatus uses components and circuits developed and studied during classes, fulfilling an important social role today. After completing the project, it is clear that the group achieved a good result, considering the implementation of a dashboard and the functioning of the circuit in LabEAD. Figure 6 depicts the web dashboard (top) and the remote laboratory bench (bottom).

**Figure 7:**
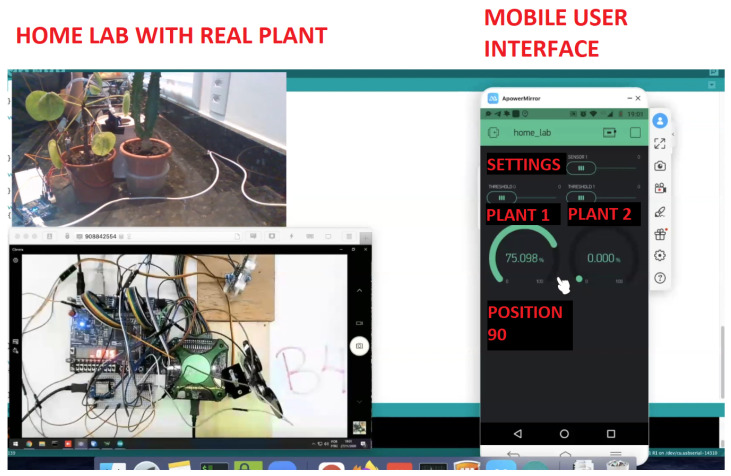
Smart Watering project.

Smart Watering: We have developed a system that waters plants as needed. This project used the home laboratory with a real plant and Arduino sensor, the remote laboratory bench, and the mobile interface. Figure 7 presents the home laboratory with a real plant and Arduino sensor (top left), the laboratory bench (bottom left), and the mobile interface (right).

**Figure 8:**
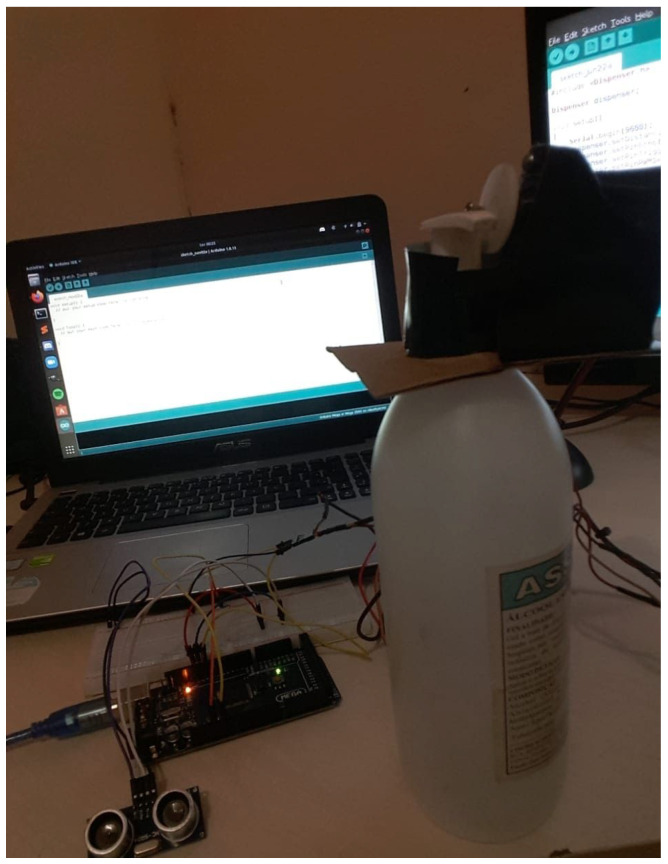
Smart Dispenser project.

Smart Dispenser: This project complements the PCS 3645—Digital Laboratory II course studies. The work proposes the development of a control system for an alcohol gel dispenser that keeps the information about customer entries and the frequency of these entries. The work was divided into modules carried out in one-week sprints. The result of the work was the implementation of a physical system, interconnected with the FPGA board of the virtual laboratory, to supply alcohol gel. Both the FPGA board and the Arduino boards in the homes of the members of the group also sent data to an API, also developed by the group. Figure 8 depicts the home laboratory (bottom left), and the dashboard (top right) developed by the students.

Safety for Puppies: With the arrival of a puppy at the student’s house, it became necessary for his family to take care that the puppy did not go to rooms in the house that were not safe for him. Thus, the project conceived by the group has the function of solving this problem, since not everyone can always check the place where the puppy is. Thus, with the circuit made, family members can be quiet if they leave the house and leave him alone, since the main door of the room will always close as he approaches, and in a second implemented mode, this main door will remain open, but the service area door will not open. In addition, in this second operating model, there is control over the other doors, other than the main room or the service area, allowing control over which rooms the puppy can enter. The results obtained were presented at the virtual fair, where we were able to demonstrate the behavior of each of the implemented modes, the free and the restricted, and we connected two Blynk cell phones so that one represented the dog and the other represented the door. With that, we manually entered the distance that would be measured by the sensor, and allowed a more fluid demonstration. In addition, with the lack of more servomotors, we represented the closing and opening of the door by means of LEDs that lit to represent the closed door and went out for the open door.

**Figure 9:**
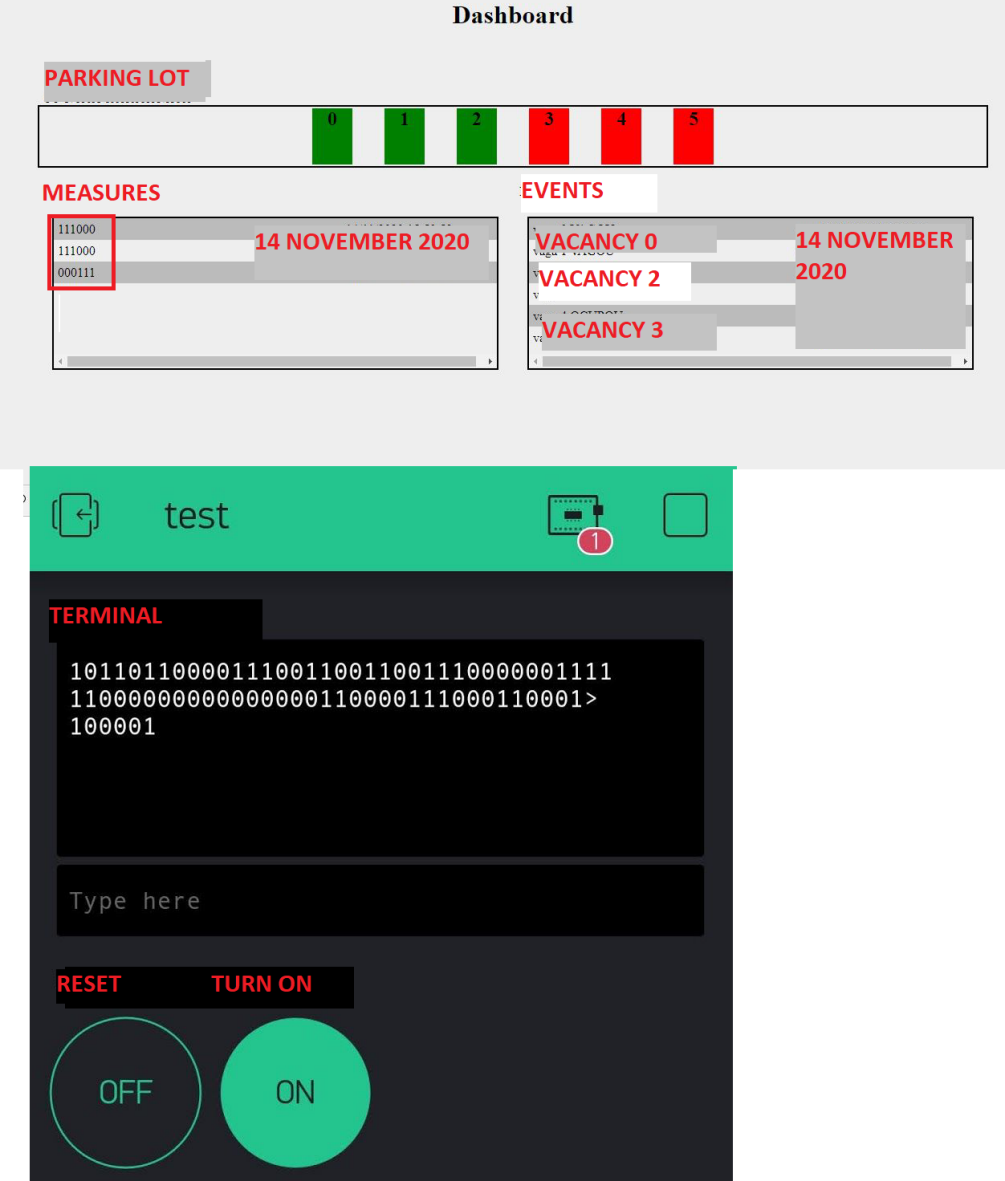
Vacancy Sensor project.

Vacancy Sensor: A parking space sensor to facilitate finding a space in a large parking lot. The project was arduous and laborious, but satisfactory in the sense that we were able to finally have something to present to the teachers. The students used a dashboard with registered events in real-time, mobile interface, and remote laboratory bench. Figure 9 depicts the dashboard with registered events in real-time (top) and mobile interface (bottom).

**Figure 10:**
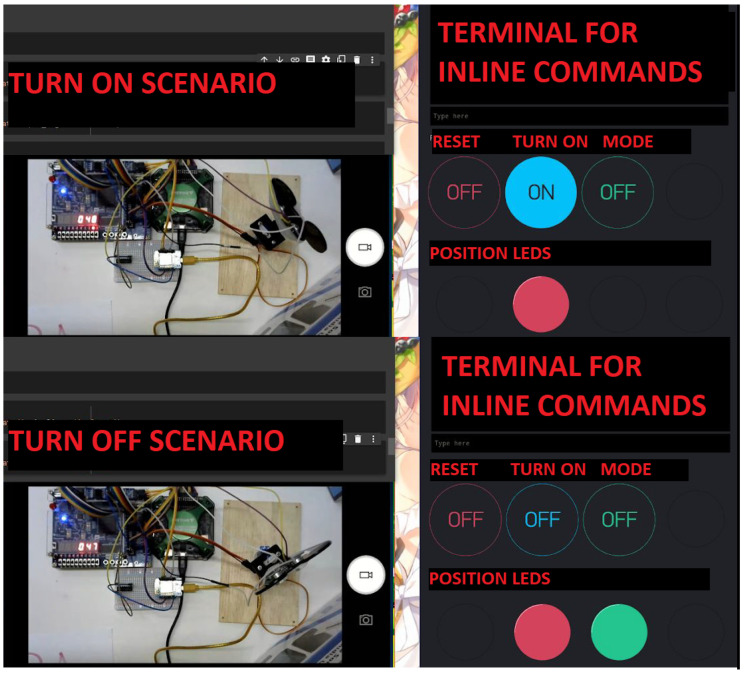
Parking Sensor project.

Parking Sensor: The project was motivated by the fact that parking sensors are a trendy option in new vehicles, useful for both novice and veteran drivers. Using the sonar module and the servomotor, whose support was developed during the first weeks of the discipline, it was sought to ensure that the final circuit had position scanning, activation of distinct proximity alarms according to distance, different operating modes (with the objective of providing the driver with better control and accuracy over measurements), plus a log with Recorders and Dashboard, storing the last two measurements, which will be used to calculate whether obstacles are approaching or moving away, and using Blynk with two cell phones per through Python scripts, to simulate a system with two inputs, one automatic from the vehicle and one manual. Figure 10 presents the laboratory bench (left) and mobile interface (right) developed by the students in two scenarios: turn on (top) and turn off (bottom).

**Figure 11:**
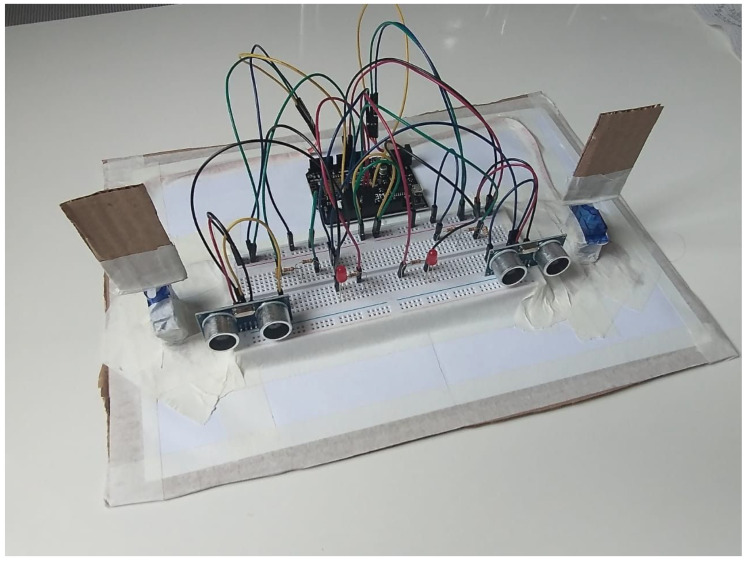
Smart Door project.

Smart Door: The problem that the project aims to solve is for people with some disability to face when trying to open a door, as they may be in unfavorable positions. The system allows the control of the doors from the cell phone and intelligently detects the door that the user wants to act. The project is partially functional since the door control modules and the central control module work correctly separately. However, when performing the integration, there is an error in the communication that has not been fixed so far. Figure 11 presents the home laboratory prototype developed by the group.

Other Projects: Table 1 presents seven projects of Digital Laboratory II in the 2020 offering with 14 students. The detailed students and teacher’s perceptions regarding the discipline offering were presented in [5].

#### 3.3.3. Third Year: Scientific Initiation

A Scientific Initiation is a short-term research project whose objective is to enable undergraduate students learn and practice the scientific method, guided by advisors.

In this case, the advisor was a Professor and the co-advisor was a Master of Science student. The advisor was responsible by providing research guidelines, and the co-advisor assisted the third-year undergraduate student in practical matters such as Arduino programming and serial communication.

The scientific initiation occurred in four months (from April to July 2020) and was comprised of:

1. Study of existing smart meter based on a one-hour interview with a specialist from the Federal University of ABC, and papers found in the literature [4,25];
2. Familiarization with tools required for the project: Fritzing [26] for hardware module design and Arduino IDE [27] for Arduino Mega programming;
3. Development of a low-cost smart meter with Arduino Mega. Tests in a home environment with fan and light bulbs. Data registration in csv file.

**Figure 12:**
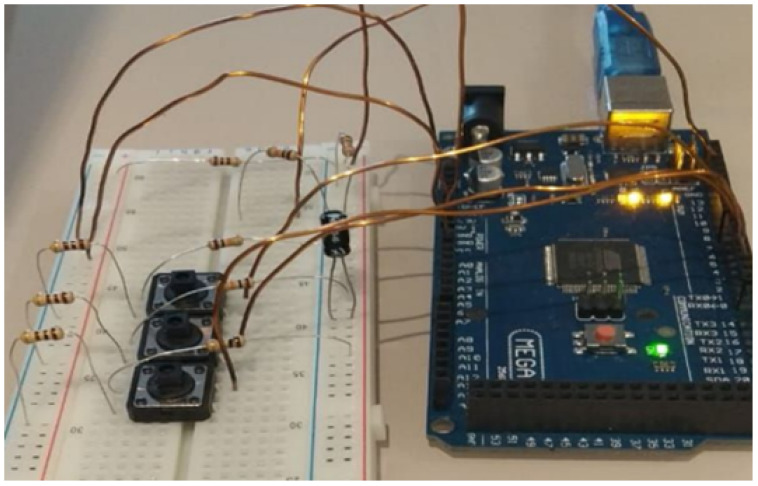
Low-cost smart meter prototype with Arduino Mega proposed in scientific initiation.

The resulting prototype is presented in Figure 12.

The whole process was performed remotely. The main challenges regard communication and physical device debugging. In some video conferences with the undergraduate student, the co-advisor used remote access to the desktop present in the undergraduate student’s home, and video streaming to verify if the prototype was connected properly. Additional project overhead was also added due to material delivery.

As a supervisor, some comments about this practice learning work: the student has done an excellent job—hardware circuits, local programming modules, and Internet connection supported considerable practical experience. Resilience was a soft skill capability developed by the student while facing challenges. The results presented in a workshop to students working as interns in companies received a good impression from the academic staff team that supports academic-industry relations.

#### 3.3.4. Fifth Year: Software Engineering Lab

The Laboratory of Software Engineering II was designed to resemble a professional environment, with a Master of Science student and Professor as Coordinators, and students grouped in squads. Each group has the freedom to choose a topic of interest to apply Software Engineering methods in the specification and documentation of such projects. Students are in their last year (fifth), and most of them work in entry-level positions in software companies.

The course consists of 15 weeks, with 15–25 min of initial presentations regarding theoretical concepts and references, and 15 min meeting with each group. While one group was in a meeting, the other could work on the project. This work method enhanced students’ autonomy and proactiveness and could be applied in face-to-face and remote alternatives.

Additional integration challenges emerging in the physical distancing context could be solved with software engineering methods such as architecture specification, simulations and mock-ups, unit tests, and integration tests. A key aspect after the course turned remote was communication, which was facilitated due to video conferencing and project management tools (e.g., Google Meet, Trello, Jira, GitHub). The students operated fully remotely from their residences, and integrated mobile applications and cloud back-end services with agile methods in an iterative manner: each week presented a functional project deliverable.

The classes were organized as follows:

1. Digital Transformation and technology trends for project motivation, expected results of projects, Osterwalder Value Proposition Canvas for project definition;
2. Software specification concepts (e.g., non-functional requisites), Collaborative development (e.g., Trello, Git);
3. Software architecture concepts and modeling;
4. Software architecture tactics for quality metrics trade-off analysis;
5. Study cases of software architectures;
6. First project documentation deliverable;
7. Microservices architecture;
8. Serverless computing;
9. Agile methods;
10. Project sprint mapping;
11. Tests and Quality metrics;
12. Software system evaluation;
13. First week of Project implementation;
14. Second week of Project implementation;
15. Final project demonstration using Google Meet, with documentation in a GitHub repository.

Table 2 presents the five projects of Laboratory of Software Engineering II in the 2020 offering with 13 students.

**Figure 13:**
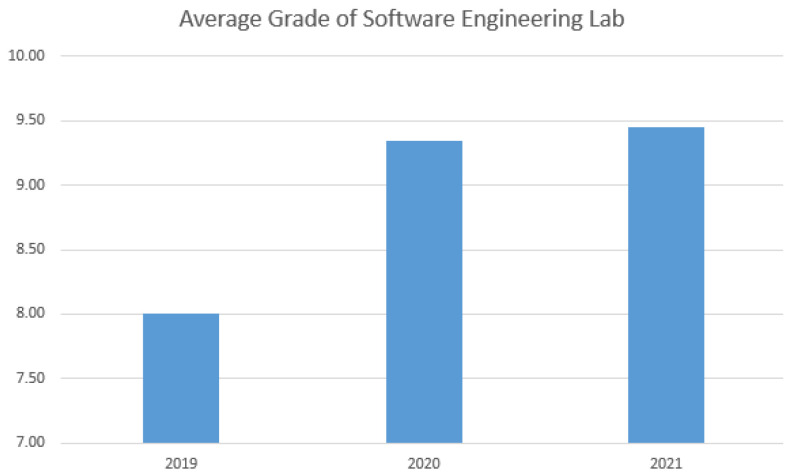
Average Student Grades for the Software Engineering Lab.

In 2021, the project’s themes were: car sharing platform, mental health app, movie recommendation assistant and privacy auditing system. Student grades of this Software Engineering course indicate no relevant differences between student performance in remote and face-to-face offerings, as one may observe in Figure 13.

#### 3.3.5. Fifth Year: Capstone Project

A smart home monitoring Capstone Project was performed from February 2020 to December 2020, with a Professor as the advisor, and the Master of Science student as the co-advisor. The fifth-year undergraduate student was responsible for project specification, implementation, and documentation.

One of the project objectives was to build a smart home monitoring infrastructure based on Arduino, Raspberry Pi and ESP8266 IoT module with built-in filesystem and Wi-Fi communication. The ESP8266 with datalogger capabilities that was used was a result of previous Capstone Project of the co-advisor and was used in this project as an accelerator. The fifth-year undergraduate student implemented the Arduino Mega interfaces with sound, motion, ultrasonic, and temperature sensors, and the middleware deployed in a Raspberry Pi device, which transferred data in real time from the testbed to a cloud back-end.

**Figure 14:**
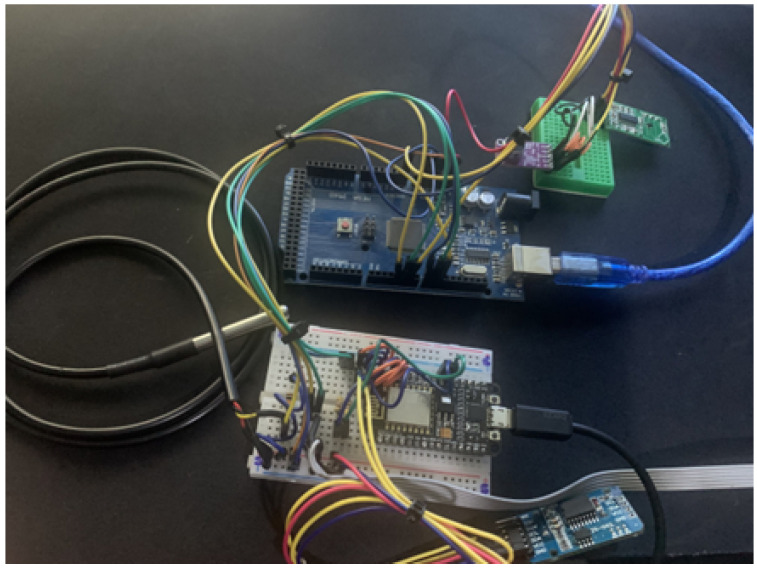
Prototype of the Smart Home monitoring Capstone Project.

An ultrasonic sensor deployed in the smart home, and the prototype with a sensing module (Arduino Mega) and a datalogger module (ESP8266) are presented in Figure 14. The data collected on the smart home testbed were integrated with the cloud by the middleware deployed on a Raspberry Pi 3.

Due to the COVID-19 pandemic, the Capstone Project was performed remotely from March to December 2020. Only initial face-to-face meetings could be conducted in February 2020. The main challenges were related to testbed prototype debugging. Remote access was performed by the co-advisor on the undergraduate student desktop connected with ESP8266 and Arduino devices.

The use of previous results from a Capstone Project (i.e., the ESP8266 datalogger module) showed the opportunity for a Master of Science co-advisor supporting future Capstone Projects. The deployment in a real smart home environment could be performed even in the social distancing context.

As a supervisor, some comments about this project: thinking and applying digital solutions to health in a Capstone Project were related to immense opportunities. The Internet of Things, cloud computing, and real-time monitoring combined to add value to people with special needs related to diabetes. The student involved constructed a tremendous professional portfolio, for sure.

## 4. Results

In this section, we present how the Sustainable Development Goals are related to the projects themes.

### Sustainable Development Goals

**Figure 15:**
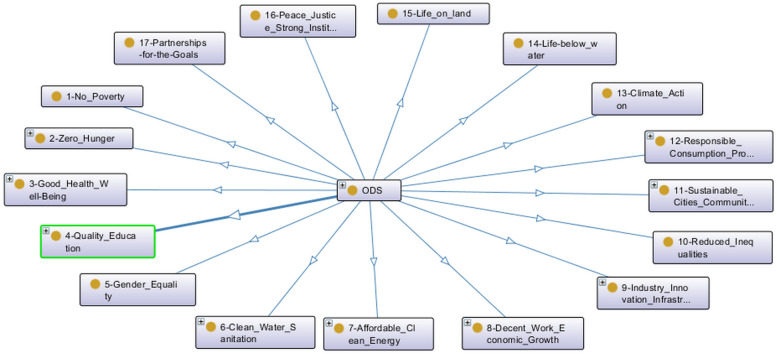
The United Nations Sustainable Development Goals in the higher level of the ontology: 1—No Poverty, 2—Zero Hunger, 3—Good Health and Well-Being, 4—Quality Education, 5—Gender Equality, 6—Clean Water Sanitation, 7—Affordable Clean Energy, 8—Decent Work Economic Growth, 9—Industry Innovation Infrastructure, 10—Reduced Inequalities, 11—Sustainable Cities Communities, 12—Responsible Consumption Production.

Based on the United Nations Sustainable Development Goals (SDG) (https://sdgs.un.org/goals, accessed on 1 February 2023), the knowledge related to the relationships among students’ projects and each SDG was modeled in an ontology using the Protégé tool [28]. The advantage of using ontologies to model this information is the graph visualizations that the Protégé tool (open-source tool developed by Stanford University School of Medicine, Stanford USA) offers. The higher level of ontology was modeled according to the 17 SDGs, as one may observe in Figure 15.

**Figure 16:**
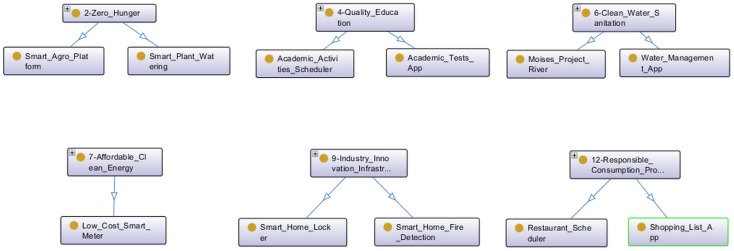
Projects related to SDGs 2, 4, 6, 7, 9 and 12.

Projects related to SDGs 2, 4, 6, 7, 9 and 12 are presented in Figure 16. A smart plant watering and agro platform may reduce food production inefficiencies, thus contributing to the SDG 2—Zero Hunger. An academic activity scheduler and academic tests app can improve education, so it is related to SDG 4—Quality Education. SDG 6—Clean Water and Sanitization has a water management app and a project related to river sanitization. The low-cost smart meter from the Scientific Initiation work relates to the SDG 7—Affordable and Clean Energy. Some innovations in the smart home infrastructure are automatic fire detection and a smart locker under the SDG 9—Industry Innovation and Infrastructure. A shopping list app and a restaurant scheduler may help to reduce wastages in supply chains regarding SDG 12—Responsible Consumption and Production.

**Figure 17:**
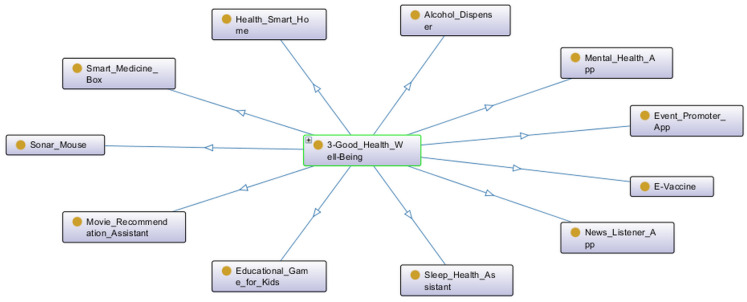
Projects related to the SDG 3.

The SDG for most projects is SDG 3—Good Health and Well-being (see Figure 17). This may be related to the COVID-19 pandemic, as health issues were focused on during this period. Some examples are: a mental health app to deal with social distancing and quarantine, a vaccination app, a smart alcohol dispenser, a smart home focused on health monitoring and an assistant for better sleep quality.

**Figure 18:**
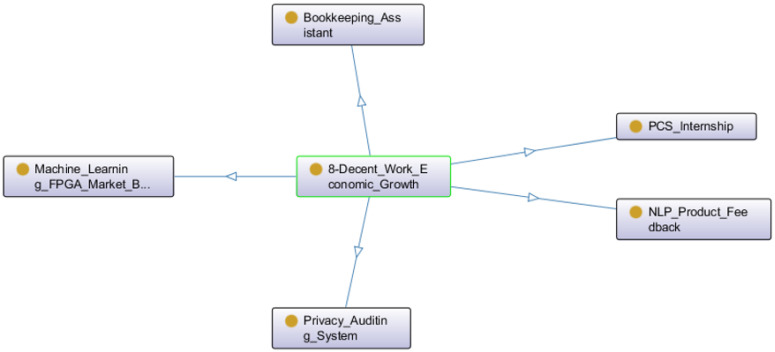
Projects related to the SDG 8.

There were five projects related to SDG 8—Decent Work and Economic Growth (see Figure 18). Some examples are a management system to find the best matches between open internship positions and students, a natural language processing application to analyze the polarization of written product feedbacks, a machine learning solution to predict market behavior and a privacy auditing system.

**Figure 19:**
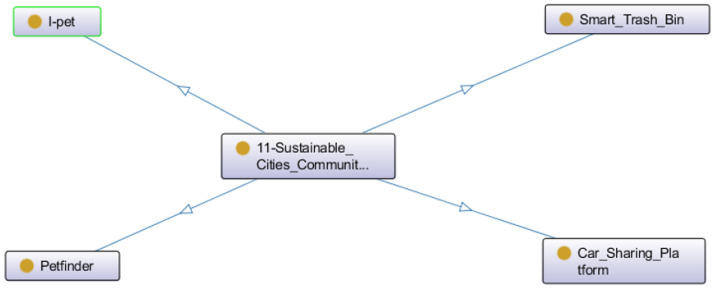
Projects related to the SDG 11.

The SDG 11—Sustainable Cities and Communities has four projects (see Figure 19). Two projects aimed to build a social network for pet owners: Petfinder to find lost pets and I-pet to share pet-related multimedia in a customized platform. The smart trash bin aims to help with waste management in sustainable cities, and the car sharing platform’s objective is to improve mobility in smart cities.

SDGs 1—Zero Poverty, 5—Gender Equality, 10—Reduced Inequalities, 13—Climate Action, 14—Life below water, 15—Life on land, 16—Peace Justice and Strong Institutions and 17—Partnerships for the Goals had no related project. These themes may be suggested in future course offerings. One hypothesis about there being no projects under SDG 5—Gender Equality is that in Computer Engineering there is a usual ratio of ten male students to one female student, and another hypothesis to explain no projects under SDG 10—Reduced Inequalitites is that most students in the specific Brazilian University where we applied this study are from higher class families. We consider that these results may vary between different Education Institutions.

## 5. Discussion

In this section, we present the comparison with related works and the response to the Research Questions.

### 5.1. Comparison with Related Works

Grodotzki et al. [29] investigated the perceptions of mechanical engineering students and manufacturing engineering professors in Germany during COVID-19. Likert-scale questions and free-text questions supported the investigation regarding different online teaching styles. Students agreed that they felt an initial lack of self-motivation, perceived the positive influence of gamification elements and enjoyed Q&A sessions more due to increased interaction. Teachers also agreed on the importance of Q&A sessions and interactions with students generally. Grodotzki et al. [29] also presented the challenges of carrying out practical activities during the pandemic, and discussed how virtual labs and remote labs might contribute to solve this issue.

The completion of 31 projects by students from our multi-case study shows that the students could engage in discipline despite the limitations of online learning during COVID-19. Thus, we argue that one possible solution for the lack of self-motivation [29] is to use Project-Based Learning in online courses.

Interactions with Q&A using the Miro tool in synchronous online classes provided opportunities for project feedback and general discussion. These interactions corroborate the perceptions from teachers and students presented by Grodotzki et al. [29].

Moreover, we also agree with Grodotzki et al. [29] regarding the challenges of laboratory courses during the pandemic. All five initiatives from first-year, third-year and fifth-year Engineering students presented in our work had practical activities. Among the virtual labs and remote labs alternatives presented by Grodotzki et al. [29], we used virtual labs for the first-year Engineering Introduction Course and the fifth-year Software Engineering Lab; we also used a remote laboratory in the Digital Electronics Lab. Another possibility was to use take-home kits, an approach we applied in the third-year scientific initiation and in the Capstone Project.

Khandakar et al. [30] used Multi-course Project-Based Learning (MPL) in an Electrical Engineering course for a senior and a capstone course. Engineering and project management skills were enhanced with the MPL approach. Furthermore, compared to the face-to-face course offering, MPL with online assessments could improve student learning outcomes during the pandemic.

Using Project-Based Learning enabled our multi-case study students to practice their technical skills (e.g., serial communication in the third-year Digital Electronics Lab course). The students could also practice their project management skills, and they could do it successfully, as shown by the delivery of 31 projects by the students. These results agree with the skills developed by Khandakar et al. [30].

Students’ and teachers’ opinions from the University of Craiova (UCV) in Romania were collected during online classes or obtained with questionnaires by Popescu et al. [31]. The results show that the transition from face-to-face to online education was incomplete over a month after the sudden forced shift to online education. Negative perceptions of students during the pandemic are related to the large workload required by online courses, the lack of interaction with colleagues and other aspects. The students also reported a deterioration in communication quality, which harmed the collaboration between students and their communication with teachers.

One course that could be completely redesigned successfully in our work was the third-year Digital Electronics Lab, but we agree with Popescu et al. [31] that it is a difficult task to accomplish. Considering the issues of lack of interaction with colleagues, less collaboration between students and worse communication with teachers, we argue that using Project-Based Learning is a way to promote communication and collaboration in online education. Students needed to specify system requirements, model its specification and develop it collaboratively in some of our case studies. Moreover, in the Q&A session, teachers could collaborate and provide meaningful feedback regarding project evolution.

### 5.2. Response to Research Questions

Regarding the research questions, the results presented in this text support the following answers:

- Answer to Research Question 1: In our work, we presented different ways in which PjBL can be applied in software and hardware courses from the Engineering curriculum during the COVID-19 pandemic. Students could explore the opportunities created by the pandemic to develop interesting IoT projects. As presented in the literature and reinforced by our work, we believe that using PjBL can mitigate the effects of emergency remote teaching, making student performance similar to face-to-face offering. Student grades of a Software Engineering course indicate no relevant differences between student performance in remote and face-to-face offerings.
- Answer to Research Question 2: Most Computer Engineering students from the Polytechnic School of the University of São Paulo in 2020 and 2021 decided to create projects related to the SDGs 3—Good Health and Well-being, 8—Decent Work and Economic Growth and 11—Sustainable Cities and Communities. Most projects were related to health and well-being, which was an expected behavior according to how health issues were brought in to highlight during the COVID-19 pandemic.

## 6. Conclusions

This article described how remote practical activities were conducted in the COVID-19 pandemic scenario for Computer Engineering at the Polytechnic School of the University of São Paulo in Brazil. The knowledge and expertise of the Internet of Things from Master of Science students (co-advisor and teaching assistant) and Professors acquired from 10 projects from 2016 to 2019 were used to propose Project-Based Learning (PjBL) approaches in undergraduate courses, Scientific Initiation and Capstone Projects in 2020 and 2021.

A total of 81 students developed a total of 31 projects. In 2020, a total of 14 projects were executed by 29 students: seven projects from the third-year´s Digital Laboratory II, five projects from the fifth-year´s Laboratory of Software Engineering II, one project in Scientific Initiation, and one Capstone Project. In 2021, a total of 17 projects were executed: 13 projects from first-year´s Engineering Introduction course and 4 projects from fifth-year´s Laboratory of Software Engineering II. The PjBL could engage Computer Engineering students in the social distancing context by presenting themes such as Sustainability, Healthcare, Smart Home (Safety and Automation), and Remote Work. Some students went beyond and integrated emerging technologies such as Machine Learning, Cloud Computing, Mobile, Internet of Things and Natural Language Processing in some projects.

It demands considerable work to build new projects with innovative applications and high technical quality based on the knowledge obtained in previous projects. However, we hope that this idea can be expanded by sharing how it was accomplished in Computer Engineering using Internet of Things technologies.

COVID-19 presented challenges to Engineering Education, but also opportunities to innovate in remote learning. Considering the efforts presented in this paper, the authors advocate that, when combined with remote labs, home labs and virtual labs, Project-Based Learning can engage students by allowing them to integrate emerging technologies into their projects and choose themes of interest. The projects could create the situations and challenges necessary for practical knowledge, initially threatened by the limitations of physical access to labs during the pandemic in 2020 and 2021.

To the best of the authors knowledge, this is one of the first works to present the relationships between the United Nations Sustainable Development Goals (SDG) and students’ projects using the Project-Based Learning (PjBL) approach. The results support the claim that the students choose project themes based on their context, so it is encouraged that future work applies this analysis to investigate if different historical and societal conditions change the relationship between SDGs and students’ project themes.

Future work may show the link between other Sustainable Development Goals (SDG) and other Engineering courses using the Project-Based Learning approach. In this study, we presented the relationship between projects from Engineering Introduction, Digital Electronics Lab, Software Engineering Lab courses and SDGs 3, 8 and 11.

## Figures and Tables

**Table 1:** Digital Laboratory II Projects.

Project Name	Description	Students
Smart Trash Bin	An IoT integrated bin, capable to communicate with a command central and track the empty fraction of its volume	2
Alcohol Dispenser	Automatic alcohol gel dispenser with a web control panel, where the owner can check the alcohol quantity inside the dispenser	2
Smart Home	IoT platform to detect fire inside houses and act against it	2
Sonar Mouse	Mouse developed with ultrasonic sensors, which receives hand movement input to move the cursor	2
Machine Learning FPGA	Neural network implemented inside the FPGA to predict stock market behaviour	2
Moisés Project	Garbage collection to be used inside rivers	2
Smart Plant Watering	An IoT platform to measure Earth humidity and automatically water plants	2

**Table 2:** Laboratory of Software Engineering II Projects in 2020.

Project Name	Description	Students
PCS_Intership	Internship system that facilitates job search, with integrated chatbot and automated digital signature to enhance usability in the remote work context.	3
EVaccine	Vaccine platform to reduce manual labor by digitalizing vaccine information. The mobile application scans codes present in the vaccine and matches location information before registration in the database. It also notifies users of ongoing vaccine campaigns.	2
Ipet	Animal monitoring platform that uses IoT camera to monitor animals (e.g., cats and dogs) at home, designed as a social network for pets.	3
NLP	Use of Natural Language Processing models to cluster and classify product feedback opinions.	1
Petfinder	Animal finding mobile application that uses image recognition algorithms to ease lost animal search. Users can look up for their lost pets, and register found pets in the platform.	4

## Data Availability

Not applicable.
